# Socially-Deviant Middle Eastern Women at a Great Risk of Poor Reproductive Health

**Published:** 2012-03-01

**Authors:** G Mohammadi, A Ramezankhani, S Amiraliakbari, H Alavi Majd, A R Farsar

**Affiliations:** 1 School of Health, Shahid Beheshti University of Medical Sciences, Tehran, Iran; 2 School of Nursing and Midwifery, Shahid Beheshti University of Medical Sciences, Tehran, Iran; 3 Department of Biostatistics, Shahid Beheshti University of Medical Sciences, Tehran, Iran; 4 School of Medicine, Shahid Beheshti University of Medical Sciences, Tehran, Iran

**Keywords:** Socially-deviant, Middle eastern, Women, Reproductive health

## Abstract

**Background:**

It is important to examine scale and scope of the reproductive health among socially-vulnerable women. The study was aimed at testing the hypothesis that as compared to general population, reproductive health is poorer among socially-vulnerable women.

**Methods:**

We enrolled 100 cases and 203 controls conducted from January 2010 to January 2011. Cases were socially-vulnerable women seeking care from Tehran’s Center for Improving Social Health of Socially-Damaged Women (Specific Center for Socially-Vulnerable Women). Controls were women referring to health centers of Tehran.

**Results:**

Mean age of cases and controls were 34.1 years and 33.8 years, respectively. Unwanted pregnancy was reported by 62.9% of cases and 33% of controls. Among socially-vulnerable women, 50.6% of abortions were reported to happen during the period in which they were using drugs. Among cases, 45.7% reported to give birth to low birth weight newborns and for controls was 11.7%. Among cases with low birth weight child, 64.9% used drug during pregnancy. Birth control was reported by 81.5% of cases and 92.8% controls. The most common method of contraception was condom among both cases (66%) and controls (31.2%). At least one screening test for cervical cancer was reported by 82.8% of controls and for 47% of cases. Mean age at first sexual intercourse was 15.7 years among cases and 20.9 years among controls. Mean Sexual Performance Scale score was lower among cases (22.3) than controls (25.9) and sexual dysfunction was more prevalent among cases (80.6%) than controls (59.1%).

**Conclusion:**

A high prevalence of reproductive health disorders was documented among socially-damaged women as compared to the women from general population.

## Introduction

Prostitution has always been there, but has not been widely accepted in many societies as a “decent” profession as it violates set norms of morality for that society. In Middle Eastern countries, there is cultural resistance to addressing the problem because the subject is largely taboo. All these countries have in common, a number of constraints that have hindered preparation of national plans of action. Often, the issue is dealt with more generally under headings such as ‘violence’ and ‘trauma’.[1] This means that there has been no regional consensus on defining prostitution in law; in some countries, for example, it is looked upon as an indecent act, in others as rape, although in some countries there is some section of the penal code that can be invoked against sexual abuse and exploitation.[2] In Islamic state, a “deviant woman,” that is, one who engages illegitimate sexual relationship holds a precarious position. On the other hand, she is pitied as a victim of social ills; on the other, whether she turns to selling sex because of dysfunctional family life, deception, or economic needs, she leads a “pathological” life and must be cured. She is at ones “socially damaged” and “socially deviant”. In the past few years, World Health Organization, the American Medical Association, International Federation of Obstetricians and Gynecologists, Royal College of Nursing, and other professional medical organizations have made statements about the public-health importance of violence against women.[2] Islamic republic of Iran policies refer to two groups of “socially deviant women.” Acutely at social-harm-risks are the highly at risk who run away from their families, have no guardian or visible means of support, or the savvy to manage their lives on their own. The category of Socially Damaged Women-Special includes those who engage in prostitution, or “women” who do not adhere to moral and social values and engage in illegitimate sex, though accrue no income in this way.[3] ”In many ways, prostitution is a very well-studied, and certainly much discussed, problem.[4] Its legal implications have been extensively analyzed. Its role in the spread of AIDS has been of equal concern.[5][6] However, there are certain issues which have been almost entirely overlooked but which are vital for a fuller understanding of the lives of women who do become prostitutes. The most conspicuous ones surround pivot of reproductive health, which have been almost entirely neglected except in the context of AIDS research.[6] It is therefore important to examine scale and score of the reproductive health among prostitutes. The authors have tested the hypothesis that as compared to general population, reproductive health is poorer among socially damaged women.

## Materials and Methods

The Study population investigated the relationship between social damage and reproductive health in 100 cases and 203 controls (at least two controls for each case). Cases were socially-vulnerable women seeking care from Tehran’s Center for Improving Social Health of Socially-Damaged Women (Specific Center for Socially-Vulnerable Women). Controls were women referring to health centers of Tehran. The study was conducted from January 2010 to January 2011.

The face-to-face interview has been conducted by using a predetermined questionnaire to be able to conduct a quantitative investigation. The questionnaire had four major components. Demographic component included data on age, marital status, and age at marriage, job, and education of participants and their husbands, when applicable. Reproductive health component included data on gestation, parity, abortion, menstruation, birth control, and cervical cancer screening. Two other components included data on sexual performance and social damage. Data on sexual performance was acquired via the Persian version of Sexual Function Scale which has been demonstrated to have acceptable external validity in Persian population.[7][8][9]

Centers for Improving Social Health of Socially-Damaged Women (Specific Center for Socially-Vulnerable Women) are centers that have recently been developed by Ministry of Health and Medical Education of Iran and any related organs with the major aim of reducing incidence of HIV/AIDS among wives of prisoners, wives of intravenous drug users, socially deviant and intravenous drug user women.

The new Health Care System in Iran was launched in 1984 to meet the nation most extensive and basic health needs throughout the country. In fact, the Health Care Hierarchy renders Primary Health Care (PHC) in Iran. The regulations of the Hierarchy intend to make health care services available to all people, to harmonize the proportion of services and manpower, to cut down expenses of the services, and to establish a referral system for health care. Currently, under the Health Care Hierarchy, the first level of the services is rendered by Health Houses in villages, and Health Posts in urban areas. Urban and Rural Health Centers in cities and villages render the second level of the services, and in case of any needs for more specialized services, patients are referred to hospitals and polyclinics, which render the third and fourth levels of the services.

Sexual dysfunction was defined as Sexual Function Scale score less than 28. Based on the current World Health Organization definition, preterm labor was ascertained in women who self-reported giving birth to a newborn before 37 weeks of gestation, counting from the first day of the last menstrual period.[10] Low birth weight was defined as a birth weight of less than 2500 grams.[11] A history of running away was defined as having run away from home or place of residence for any number of reasons under the age of 18. This may include situations where it is suspected that the child has been coerced or encouraged to leave home by a third party (not their parent). This may also include children that have stayed away at least one night or for more than 24 hours in the past or who are in the care of a child welfare agency and are believed to have voluntarily left their placement. Definition of running away involves being voluntarily absent from home at least overnight without permission from a parent or caretaker, or legal guardian.[12] The definition of abortion was the termination of pregnancy by any means before 20 weeks based upon the date of the first day of the last normal menses. Abortion was defined as spontaneous when abortion occurs without medical or mechanical means to empty the uterus or induced abortion when the medical or surgical termination of pregnancy before the time of fetal viability. History of stillbirth was ascertained in women who self-reported no signs of life were present in a child at or after they had given birth to. Cesarean delivery was defined by self-reporting. Menstrual bleeding was considered irregular if it occurs more frequently than every 21 days, lasts longer than 7 days or is heavy. Irregular bleeding also included infrequent periods, including menstrual intervals greater than 35 days, skipped months or absent periods.[13]

For descriptive statistics, data were presented as mean (SD) and frequency (percent) for continuously and categorically distributed variables, respectively. Significance of differences were tested by either χ(2) test (were appropriate) or Mann–Whitney test for categorically and ordinally distributed variables, respectively. The statistical significance level was set at a two-tailed type I error of 0.05. All statistical analyses were performed using PASW Statistics 18 (SPSS Inc., Chicago, IL, USA).

We certify that all applicable institutional and governmental regulations concerning the ethical use of human volunteers were followed during this research. Informed written consent was obtained from all participants and the Ethical Committee of Shahid Beheshti University (M.C.) approved this study.

## Results

General characteristics of participants were demonstrated in Table 1. Mean age of cases and controls were 34.1 (±7.7) years and 33.8 (±9.7) years, respectively, (p=0.72). Divorce was reported by 48.9% of cases and 4.4% of controls (p<0.001). Age at first marriage was 16.5 (±4.1) years for cases and 20.8 (±3.9) for controls (p<0.001). Mean age of husbands at first marriage was 24.7(±6.2) years among cases and 25.9 (±4.3) among controls (p=0.130). As is shown in Table 1, cases and their husbands were less educated that their control counterparts (p<0.001). Prevalence of unemployment was observed to be higher among vulnerable women (52.2%) than among general population (50%), (p<0.001). At least one run-way from home was reported by 39% of cases. 34.5% of vulnerable women who self-reported runaway from home also reported to be sexually abused during their run-away. Drug abuse was reported by 96.9% of cases and 55.1% were using drugs at the time of interview. Mean age at first drug use was reported to be 20.3 (8.7) years among cases. Mean age at first pregnancy was 19.4 (±5.3) years among cases and 22.9 (±4.5) years among controls (p<0.001).

**Table 1:** General characteristics of cases and controls.

		**Controls**		**Cases**		***P* value**
		**Frequency**	**Percent**	**Frequency**	**Percent**	
**Age (years)**	<25	14	6.9	27	27	<0.001
	25-29	51	25.1	13	13	
	30-34	53	26.1	8	8	
	35-39	36	17.7	19	19	
	40-44	27	13.3	19	19	
	≥45	22	10.8	14	14	
**Job **	Unemployed	162	79.8	57	57	0.001
	Manual worker	6	2.9	15	15	
	Office worker	30	14.8	7	7	
	Huckster	5	2.5	21	21	
**Husbands’ job**	Unemployed	2	0.99	25	27.2	0.001
	Manual worker	15	7.4	15	16.3	
	Office worker	80	39.5	6	6.5	
	Other	106	52.2	46	50	
**Education **	Illiterate	4	2.0	17	17	<0.001
	Elementary	12	5.9	26	26	
	Guidance school	27	13.3	30	29	
	High school	105	51.7	24	24	
	Academic	55	27.1	3	3	
**Husbands’ education**	Illiterate	0	0	9	9.8	<0.001
	Elementary	16	7.9	34	37	
	Guidance school	45	22.2	13	14.1	
	High school	85	41.9	28	30.4	
	Academic	57	28	8	8.7	

Table 2 compares elements of reproductive health between cases and controls. Cases reported more pregnancies (98.5%) than did controls (89.0%) (p<0.001). Unwanted pregnancy was reported by 62.9% of cases and 33% of controls (p<0.001). Among socially-vulnerable women, 50.6% of abortions were reported to happen during the period in which they were using drugs. Among cases, 45.7% reported to give birth to a low birth weight newborn and the corresponding figure among controls was 11.7% (p<0.001). Among cases with low birth weight child, 64.9% reported to use drug during pregnancy. Birth control was reported by 81.5% of cases and 92.8% of controls (p<0.001). The most common method of contraception was condom among both cases (66%) and controls (31.2%). At least one screening test for cervical cancer was reported by 82.8% of controls while only 47% of cases reported ever having been screened for cervical cancer (p<0.001). Mean age at first sexual intercourse was 15.7 (±3.8) years among cases and 20.9 (±3.8) years among controls (p<0.001). Mean Sexual Performance Scale score was lower among cases (22.3) than controls (25.9) and sexual dysfunction was more prevalent among cases (80.6%) than controls (59.1%), (p<0.001).

**Table 2:** Comparison of reproductive health elements between cases and controls.

	**Controls**		**Cases**		***P* value**
	**Frequency**	**Percent**	**Frequency**	**Percent**	
**Irregular bleeding **	34	18.6	30	46.2	<0.001
**At least one gestation **	200	98.5	89	89.0	<0.001
**Unwanted pregnancy**	66	33.0	56	62.9	<0.001
**History of life birth**	197	98.5	81	91.0	0.004
**History of abortion **	44	22.0	45	50.6	<0.001
**History of Low birth weight**	23	11.7	37	45.7	<0.001
**History of preterm labor**	6	26.1	9	24.3	<0.001
**History of Cesarean delivery**	96	48.7	26	32.1	0.004

## Discussion

In this case-control study, the authors documented that social-damage was associated with poor reproductive health. The authors observed that low birth weight, premature delivery, unwanted pregnancy, induced abortion, and sexual dysfunction were all more prevalent among socially-damaged women than general population. Socially-damaged women were less likely to be educated, take cervical cancer screening test or use any contraceptive method.

To explore the prevalence and risk factors of female sexual dysfunction (FSD) in Iran, Safarinejad et al.[14] investigated data on a total of 2626 women aged 20-60 years using a self-administered questionnaire. They have reported 31.5% (759) of the general population females to be involved with FSD. The prevalence increased with age, from 26% in women aged 20–39 years to 39% in those >50 years (tested for trend, p<0.001). Thirty-seven percent reported orgasm disorders (OD), 35% desire disorders (DD) and 30% arousal disorders (AD), all of which increased significantly with age. Pain disorders were reported by 26.7%, occurring most frequently in women aged 20–29 years. The reported disorders were found to be inversely correlated with the educational level, and marriage age (<18 years). No significant differences were detected in smoking history, the presence of previous pelvic surgery and contraception methods used. A history of psychological problems, married status, low physical activity, chronic disease, multiparity, menopause status, and spousal erectile dysfunction were significantly associated with FSD.

As compared to general population, socially-damaged Persian women married at younger ages. The marriages also ended up with higher frequency among socially damaged women. Marriage has been reported to be less frequent among socially-damaged women of other population.[15][16] This might have, at least in part, contributed to the higher frequency of divorce observed in the current study[17][18][19] and possible explanations could be lower educational estate or the socially-damaged women and their husbands.[19] For Persian women, marriage is the only culturally, morally, and legally acceptable situation in which sexual activity is allowed.[20][21] This could have possibly predisposed young girls to early marriage; the marriage, of which most aspects other than sex has been neglected and thus, have been subjected to failure and divorced.[22][23][24][25] Finally, the finding that unemployment was more prevalent among husbands of socially-damaged women could have not escaped our notice in this regards. It has been shown that men's unemployment doubles the risk of divorce in the first 5 years of marriage, whereas wife's unemployment had no effect as long as her husband was employed.[26][27] Marriage (sexual activity) at young age made women more likely to have pregnancy and incidence of unwanted pregnancy was unacceptably high among teenage girls.[28][29][30] More than half of the girls first intercourse has been reported to be unplanned and, therefore, unprotected.[31] Unwanted pregnancies in their own turns, made induced abortion more likely to be attempted.[32][33][34] During early stages of unplanned pregnancies women were less likely to aware of their pregnancies. This could possibly render them more likely to be exposed to risk factors and potentially increase the risk of low birth weight and premature delivery.[35][36]

History of Cesarean section was less frequently reported by socially-damaged women than women from general population. This may be an indirect indication of lower accessibility to health services in this subgroup of women. The gap between menarche and marriage has been shown to be a risk factor for pre-marital sexual activity without access to health services.[37] The same implication could have been applicable to the finding that socially-damaged women were less likely to use contraceptive methods or take cervical cancer screening test.

Social-damage was associated with low Sexual Function Scale score and high prevalence of sexual dysfunction. Several factors have been reported to have contributing role; few examples are sexual trauma, low education, divorce, life style, chronic illness, and previous experiences like domestic violence or rape, substance abuse.[38] The authors are aware of only one study that have specifically assessed sexual difficulties in sex workers; Munasinghe et al., showed that, the prevalence of sexual difficulties, other than desire was similar to those of non-sex workers. Compared with non-sex workers, a higher percentage of women working in the sex industry experience a lack of interest in sex with their private partners and fewer sex workers reported being distressed about their personal sex lives. Sex workers were equally emotionally satisfied and reported similar levels of physical pleasure.[39] Their study sample coming from highly regulated sex industry might have contributed the differences with our findings.[39] It has been shown women working in licensed brothels came from a wide range of backgrounds and circumstances and hold varying attitudes towards working in the sex industry. Many women have been reported to have actively chosen this occupation based on the financial rewards and flexibility it offers. Like many other women, these women are striving for further education or training, supporting families, or pursuing reach financial goals. Many of these women do not express a desire to leave the sex industry. As the authors themselves stated it is possible for that the women who remained working in the sex industry were those who did not have adverse effects from sex work.[40]

Running away from home has been previously reported to be associated with poor reproductive health. Runaway street youths are at greater risk for a wide variety of medical problems and of health compromising behaviors including suicide and depression, prostitution, and drug use.[41] The authors observed a high prevalence of run-away among socially-damaged women.

It can not be more emphasize that we need to ensure that the socially-damaged women are protected and have access to health and medical services. Social damage is a reproductive health issue. With or without legalization, reproductive health services designed to cater for the deviant women could be set up to ensure that it is accessible and that services are offered in a nonjudgmental manner and free of charge.

The authors used face-to-face interview, a method that increased validly and accuracy of data gathering. All questionnaires were completed by the same person. This approach would eliminate inter-rater bias. Socially-damaged women were those who were seeking care from centers for Improving Social Health of Socially-Damaged Women. It is possible that we under-estimate the disorders since the reproductive health might have been even poorer among those who had no access to these centers; although, no data to show if these centers have improved health estates of their clients. Collecting information from this group was not easy. It was difficult to follow the structured interview guide with this group of women who have had chaotic lives., reluctance to acknowledge that women people may engage in sexual activity, or that commercial sex exists in the region, make it extremely difficult assess and therefore improve reproductive health among this sub-group. Finally, cases and controls were not matched.

The authors documented a high prevalence of reproductive health disorders among socially damaged women as compared to the women from general population. Considering that our study sample included socially-damaged women who were seeking care from centers for Improving Social Health of Socially-Damaged Women, it is possible that the reproductive health estate is even worse among socially-damaged women who had no access to these centers.
